# Life-Saving Intracranial Mechanical Thrombectomy for Acute Ischemic Stroke in Pregnancy: Balancing Maternal and Fetal Risk

**DOI:** 10.14740/jmc5335

**Published:** 2026-06-03

**Authors:** Eugen Enesi, Vojsava Leka, Gentian Huti, Arben Rroji, Stela Dodaj, Klodian Caci, Asead Abdyli, Driola Hoxha, Oliatina Demiri, Besnik Filaj, Sotiraq Lako, Indrit Temali, Denisa Bajraktari, Aida Agastra, Filadelfo Coniglione, Mirel Grada, Mentor Petrela, Rudin Domi

**Affiliations:** a Department of Neurosurgery and Interventional Neuroradiology, American Hospital 3, Tirana, Albania; b Department of Clinical Sciences, Faculty of Medical and Technical Sciences, University of Medicine, Tirana, Albania; c Department of Anesthesiology and Intensive Care Medicine, American Hospital 3, Tirana, Albania; d Department of Onco-Hematology, American Hospital 3, Tirana, Albania; e Department of Cardiology, American Hospital 3, Tirana, Albania; f Department of Obstetrics-Gynecology, American Hospital 3, Tirana, Albania; g Department of Clinical Science and Translational Medicine, Tor Vergata University of Rome, Rome, Italy; h Department of Surgery, Faculty of Medicine, University of Medicine, Tirana, Albania

**Keywords:** Stroke, Pregnancy, Mechanical thrombectomy, Anesthesia, Intensive care unit, Thrombophilia

## Abstract

Stroke during pregnancy is uncommon but poses a significant threat to both mother and fetus, requiring rapid, carefully balanced interventions. We describe a 46-year-old woman at 24 weeks’ gestation who presented with sudden speech difficulties, facial droop, and weakness on one side of her body. Imaging revealed an acute right middle cerebral artery occlusion, and extensive evaluation ruled out cardioembolic, thrombophilia, and vascular causes. She underwent urgent mechanical thrombectomy under monitored anesthesia care, achieving partial revascularization without complications. Her neurological function improved progressively, and both maternal and fetal outcomes were favorable. This case highlights the complexity of managing acute stroke in pregnancy, where timely decision-making must integrate standard stroke protocols with pregnancy-specific considerations, including imaging, anesthesia, and multidisciplinary care. Sharing such experiences is essential to expand knowledge on the safety and effectiveness of endovascular interventions in this rare but critical setting.

## Introduction

Stroke remains a leading cause of mortality and morbidity worldwide, affecting both sexes and individuals across all age groups, with an increasing impact observed not only in the elderly but also among younger populations [1]. According to the Global Stroke Fact Sheet 2025 by World Stroke Organization, stroke remains a major global health burden, causing an estimated 12 million new cases and over 7 million deaths annually, making it the second leading cause of death worldwide. While age-standardized rates have modestly declined in some regions, absolute numbers have risen sharply since 1990, driven by population growth and aging. Ischemic stroke accounts for 65% of cases, with intracerebral and subarachnoid hemorrhage comprising most of the remainder. Stroke increasingly affects younger adults, with nearly one-third of deaths occurring before age 70, and the burden is disproportionately high in low- and middle-income countries, reflecting ongoing disparities in prevention and care [2]. Stroke during pregnancy is a rare but serious complication with significant clinical implications. Miller reports that the overall incidence of pregnancy-associated stroke is roughly 30 per 100,000 pregnancies, exceeding the risk observed in age-matched non-pregnant women. Both ischemic and hemorrhagic strokes occur, with ischemic events representing a substantial share. Although uncommon, these strokes are associated with notable maternal morbidity and mortality, with death rates estimated at about 1–2 per 100,000 pregnancies. The highest risk period is during the late third trimester and early postpartum, when pregnancy-related physiological, hemodynamic, and hypercoagulable changes are most pronounced [3, 4].

Despite advances in acute stroke management, evidence regarding the diagnosis and treatment of ischemic stroke during pregnancy remains limited, as pregnant patients are often excluded from major clinical trials and guideline-defining studies. Challenges therefore persist regarding imaging strategies, anesthetic management, maternal–fetal safety, radiation exposure, and timely reperfusion therapy. Consequently, reporting such cases provides valuable clinical experience to support decision-making and future guideline development in this high-risk population.

We present a case of acute ischemic stroke during pregnancy, in which clinical assessment and magnetic resonance imaging (MRI) examination established the diagnosis, followed by urgent mechanical thrombectomy performed under monitored anesthesia care (MAC), resulting in favorable maternal and fetal outcomes. This case highlights that, despite the unique diagnostic, anesthetic, and maternal–fetal management challenges associated with pregnancy, timely multidisciplinary intervention can achieve successful neurological and obstetric outcomes, emphasizing the importance of early recognition and rapid reperfusion therapy in pregnant patients with acute ischemic stroke.

## Case Report

### Investigations

A 46-year-old pregnant woman, at 24 weeks of gestation in her first ongoing pregnancy, presented to the emergency department with acute onset of neurological symptoms. She reported difficulty speaking, facial asymmetry, and impaired control of the left-sided limbs, with symptom onset approximately 2 h prior to admission.

Following initial stabilization, comprehensive neurological and obstetric evaluations were performed, and urgent neuroimaging was arranged.

On neurological examination, the patient was fully conscious without cognitive deterioration and showed no signs of meningeal irritation. The pupils were equal, round, and reactive to light, with preserved extraocular movements. Motor dysarthria was present, while the gag reflex remained intact.

Further examination revealed left-sided central facial paralysis, left hemi hypoesthesia, and left hemiparesis, with motor strength graded at 2–3/5. A positive Babinski sign was elicited on the left side. Deep tendon reflexes were preserved, and mild motor ataxia was observed. The National Institutes of Health Stroke Scale (NIHSS) score at admission was 10, Glasgow Coma Scale (GCS) score of 15.

Obstetric evaluation demonstrated a normal fetal heart rate, posterior placental insertion, and a three-vessel umbilical cord. Fetal biometric measurements were consistent with the gestational age, with no abnormalities detected. The patient weighed 69 kg, measured 162 cm in height (body mass index (BMI) 26.3 kg/m^2^), and this was her first pregnancy following natural conception after several previous spontaneous miscarriages. Antenatal care had been regularly and uneventfully completed until the stroke event. She had no previous history of hypertension, including during hospital admission and throughout the hospital stay.

### Diagnosis

MRI, including T2-weighted, fluid-attenuated inversion recovery (FLAIR), and diffusion-weighted imaging (DWI) with apparent diffusion coefficient (ADC) mapping, demonstrated an acute ischemic lesion in the right frontal–insular region (Figs. 1, 2).

**Figure 1:**
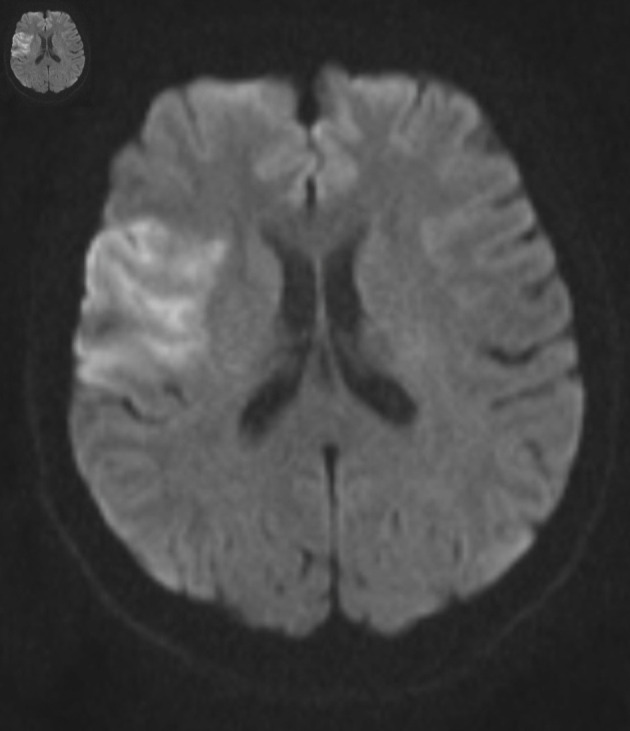
Diffusion-weighted MRI (DWI) demonstrates signal restriction in the right frontal-insular region consistent with acute ischemic stroke. DWI: diffusion-weighted imaging; MRI: magnetic resonance imaging.

**Figure 2:**
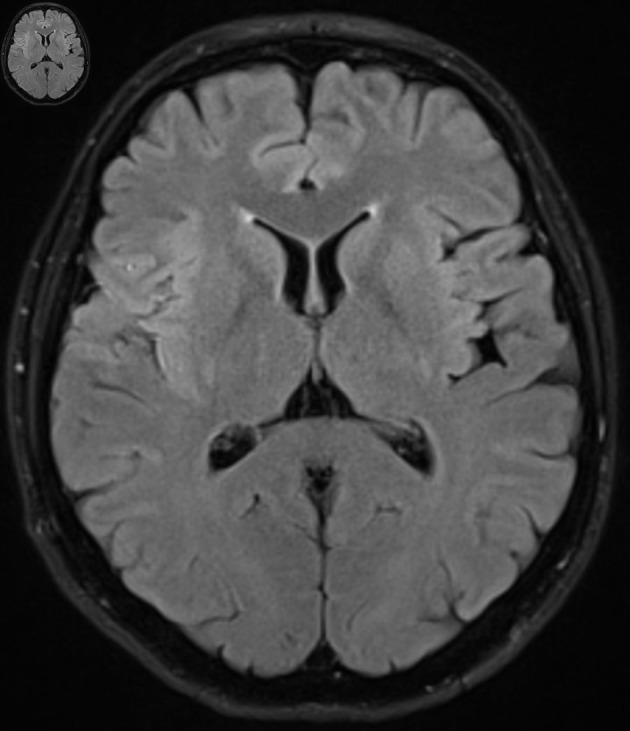
FLAIR MRI demonstrates a right frontal-insular hyperintensity consistent with an ischemic lesion. FLAIR: fluid-attenuated inversion recovery; MRI: magnetic resonance imaging.

Magnetic resonance angiography revealed absence of flow in the right M2 segment of the middle cerebral artery (MCA) (Fig. 3).

**Figure 3:**
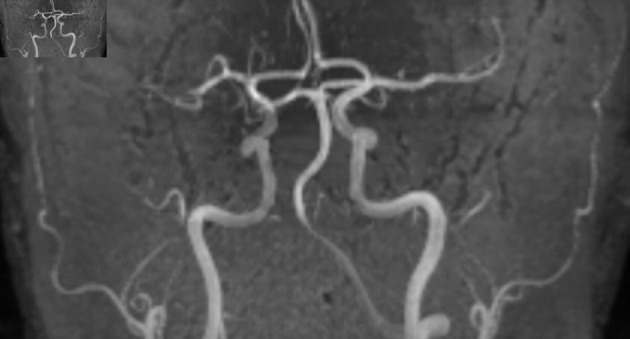
MR angiography demonstrates absence of flow in the right M2 segment of the middle cerebral artery. MR: magnetic resonance.

Based on the clinical presentation and imaging findings, a diagnosis of acute ischemic stroke in the right MCA territory was established.

The patient’s medical history was significant for multiple spontaneous abortions and hypothyroidism, for which she was receiving daily treatment with 75 µg of levothyroxine. Cardiological evaluation with transthoracic echocardiography revealed normal cardiac structure and function, with only mild pregnancy-related physiological mitral regurgitation and no evidence of interatrial or interventricular shunting. A bubble test performed during transthoracic echocardiography was negative. These findings were further confirmed by transesophageal echocardiography and a repeat bubble test, effectively excluding a cardioembolic source.

Further vascular assessment with Doppler ultrasound of the carotid arteries and deep veins of the lower extremities showed no abnormalities.

Given the history of multiple spontaneous abortions, antiphospholipid syndrome was suspected. However, the diagnosis remained presumptive, as it was not confirmed by laboratory testing. The patient was advised to repeat the relevant laboratory investigations for further evaluation. An extensive hematological and coagulation workup was also performed to evaluate thrombophilia and antiphospholipid syndrome. Results were within normal limits, including complete blood count, hemoglobin electrophoresis, prothrombin time (PT), activated partial thromboplastin time (aPTT), and levels of vitamin B12, ferritin, antithrombin III, protein C, and protein S. Immunological testing for immunoglobulin (Ig)G and IgM anticardiolipin antibodies and anti-β2 glycoprotein antibodies was negative.

### Treatment

Given the diagnosis, the patient underwent urgent mechanical thrombectomy under MAC via femoral artery access. Intra-procedural angiography confirmed an occlusion of the right superior M2 branch of the MCA (Fig. 4). Revascularization was achieved using a combined technique of aspiration thrombectomy and stent retriever deployment, resulting in restoration of blood flow with a Thrombolysis in Cerebral Infarction (TICI) 2b grade (Fig. 5).

**Figure 4:**
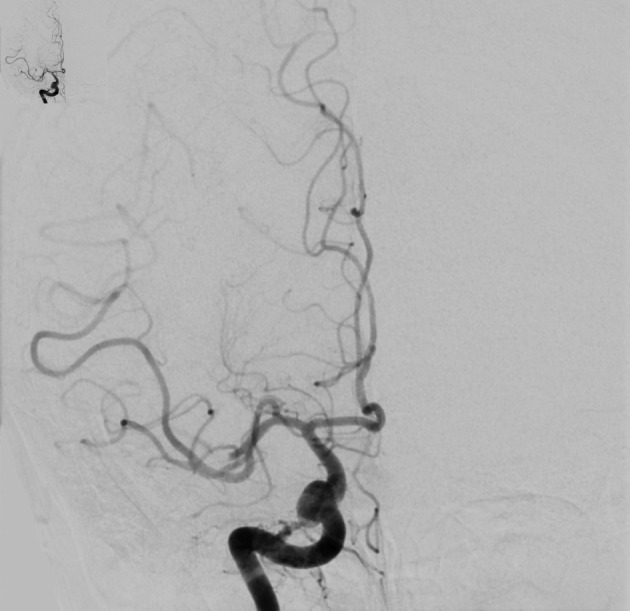
Digital subtraction angiography (DSA) confirms complete occlusion of the right superior M2 segment, with absent flow in its vascular territory.

**Figure 5:**
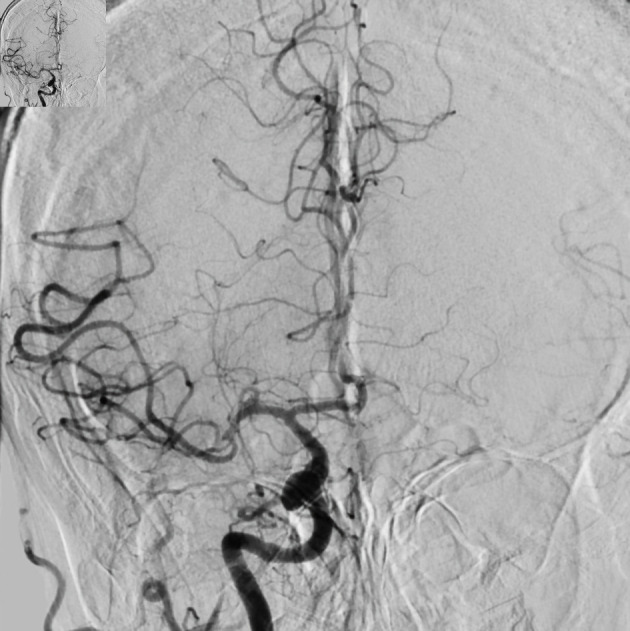
Digital subtraction angiography (DSA) performed after mechanical thrombectomy demonstrates TICI 2b recanalization of the right superior M2 segment. TICI: Thrombolysis in Cerebral Infarction.

### Follow-up and outcomes

The procedure was uneventful, and the patient was subsequently transferred to the intensive care unit (ICU) for close neurological and hemodynamic monitoring.

Follow-up brain MRI with DWI, performed 24 h after thrombectomy, showed no new ischemic lesions, apart from the lesion identified on admission (Fig. 6).

**Figure 6:**
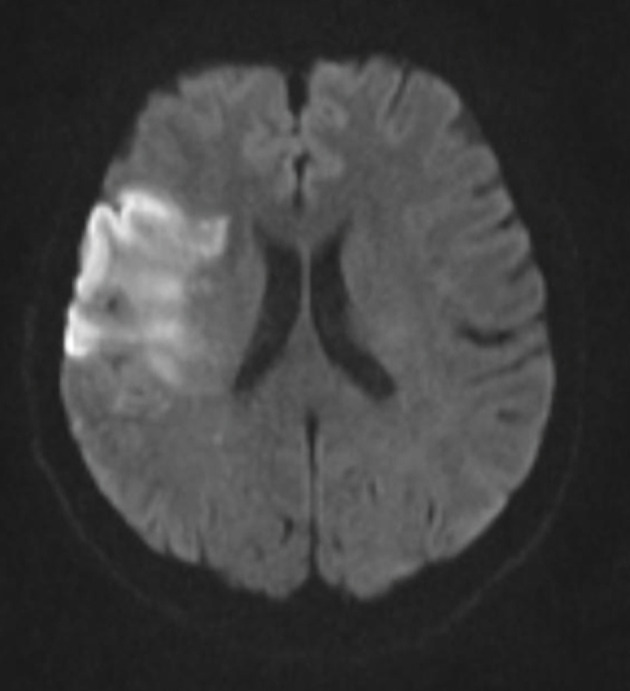
Diffusion-weighted MRI (DWI) performed 24 h after thrombectomy demonstrates frontal-insular signal restriction, consistent with the evolving infarct, without evidence of new ischemic events during or after the procedure. DWI: diffusion-weighted imaging; MRI: magnetic resonance imaging.

Due to the favorable outcome and progressive clinical improvement, she was transferred to the general ward the following day. Over the course of hospitalization, her neurological deficits showed gradual improvement. After 1 week at discharge, the patient demonstrated clinical improvement, with an NIHSS score of 3. She was prescribed low-molecular-weight heparin (LMWH) for thromboprophylaxis during pregnancy, along with additional therapy as recommended by endocrinology and gynecology specialists. Continued multidisciplinary follow-up was advised.

The pregnancy remained uneventful until term. During follow-up after mechanical thrombectomy, gynecological evaluations demonstrated a closed uterine cervix, normal fetal heart rate, and normal amniotic fluid volume throughout the remainder of the pregnancy. At 39 weeks of gestation, the patient underwent cesarean section under spinal anesthesia with careful hemodynamic management aimed at avoiding hypotension. A healthy newborn with normal Apgar scores of 9 and 10 at 1 and 5 min, respectively, was delivered without reported perinatal complications. Both mother and newborn were discharged in good clinical condition. Maternal neurological status remained stable, with continued functional recovery during follow-up, supporting the favorable outcomes observed in this case.

## Discussion

### Pregnancy-related coagulation changes

Pregnancy is characterized by a physiological hypercoagulable state that functions as a protective mechanism against excessive bleeding during delivery, but at the same time increases the risk of thrombosis. This state is marked by elevated levels of procoagulant factors, particularly factors VII, VIII, X, and fibrinogen, together with a reduction in natural anticoagulant activity, most notably decreased protein S levels. In parallel, fibrinolysis is suppressed, further promoting clot stability. Collectively, these changes shift the hemostatic balance toward enhanced thrombin generation and fibrin formation, creating a prothrombotic milieu that, while beneficial for hemostasis at parturition, predisposes pregnant women to thromboembolic complications [4, 5]. Thrombotic complications during pregnancy are predominantly venous, with deep vein thrombosis and pulmonary embolism representing the most common and clinically significant events and remaining leading contributors to maternal morbidity and mortality [6, 7]. Less frequently, thrombosis involves the cerebral venous system, leading to cerebral venous sinus thrombosis (CVST) with variable neurological manifestations. CVST is a rare subtype, with a baseline incidence of approximately 3–5 cases per million annually, which increases during pregnancy and the puerperium. Up to one-fifth of cases are pregnancy-related, with the postpartum period representing the highest-risk window. Reported incidence varies widely across regions, ranging from about 1 in 2,500 to fewer than 1 in 10,000 deliveries, with higher rates observed in low-resource settings. Thus, although relatively uncommon, pregnancy-associated thrombosis represents a meaningful increase in risk driven by both physiological and obstetric factors [8, 9]. Arterial thrombotic events, including ischemic stroke and myocardial infarction, are rare but clinically important, particularly in women with underlying risk factors. Acute myocardial infarction (AMI) during pregnancy is an uncommon yet potentially life-threatening condition, occurring in approximately 1 in 16,000 pregnancies and associated with substantial maternal and fetal risk. Its pathophysiology differs from that in the general population, with spontaneous coronary artery dissection (SCAD) emerging as the leading cause, followed by atherosclerosis and coronary thrombosis. Risk is further amplified by advanced maternal age, hypertension, diabetes, smoking, and underlying thrombophilia, in addition to pregnancy-related physiological changes. Diagnosis may be challenging due to symptom overlap with normal pregnancy and concerns regarding imaging. Management requires an individualized, multidisciplinary approach that balances maternal stabilization and timely reperfusion with fetal safety, incorporating appropriate pharmacologic therapy, percutaneous or surgical revascularization when indicated, and careful planning of delivery and postpartum monitoring [10]. Moreover, underlying prothrombotic disorders such as antiphospholipid syndrome and inherited thrombophilia play a critical role by increasing both maternal thrombotic risk and the likelihood of adverse pregnancy outcomes, including placental dysfunction and fetal loss [11].

### Specific considerations of ischemic stroke during pregnancy

Although uncommon, ischemic stroke in pregnancy is an increasingly recognized clinical concern, with an estimated incidence of 10–30 per 100,000 pregnancies and a higher occurrence in the peripartum and early postpartum periods, largely driven by pregnancy-associated hypercoagulability [12]. As highlighted by Pacheco et al, management principles should largely mirror standard acute stroke care, while being adapted to maternal–fetal considerations [4]. Rapid recognition and immediate neuroimaging are essential. Non-contrast head computed tomography (CT) should not be delayed, as fetal radiation exposure is minimal compared with the risks associated with diagnostic delay. Reperfusion remains the cornerstone of treatment: intravenous alteplase may be administered within the established 4.5-h window following careful individualized assessment, as pregnancy is not an absolute contraindication, while mechanical thrombectomy is indicated in selected patients with large-vessel occlusion. Anticoagulation is generally avoided in the hyperacute phase due to the increased risk of hemorrhagic transformation, whereas antiplatelet therapy is used for secondary prevention. Equally important is meticulous supportive care, including optimization of blood pressure, glycemic control, normothermia, and fluid balance, all of which influence maternal and fetal outcomes [13]. The mechanisms underlying maternal stroke are multifactorial, reflecting the interplay between pregnancy-related physiological changes and traditional vascular risk factors. A prothrombotic state driven by increased coagulation and reduced fibrinolysis, significantly elevates thrombotic risk, particularly in late pregnancy and the postpartum period. Hemodynamic adaptations, including increased blood volume and cardiac output, may contribute to endothelial dysfunction and susceptibility to both ischemic and hemorrhagic events. Hypertensive disorders, especially preeclampsia and eclampsia, are major contributors through impaired cerebral autoregulation and increased hemorrhagic risk. Additional mechanisms include cardio-embolism (e.g., peripartum cardiomyopathy), cervical artery dissection, and pregnancy-specific conditions such as CVST and reversible cerebral vasoconstriction syndrome (RCVS), with rarer causes including amniotic fluid and paradoxical embolism [13]. Management generally follows standard stroke protocols but requires important pregnancy-specific adaptations to balance maternal benefit and fetal safety. Reperfusion therapies including intravenous thrombolysis and mechanical thrombectomy should not be withheld when indicated, although individualized risk–benefit assessment remains essential due to potential maternal and obstetric bleeding. Medication choice is critical: when anticoagulation is required, heparins are preferred, while warfarin and most direct oral anticoagulants are typically avoided because of fetal risks [14, 15]. Blood pressure control should rely on pregnancy-safe agents (e.g., labetalol, nifedipine), avoiding teratogenic drugs such as angiotensin-converting enzyme (ACE) inhibitors [16]. In cases associated with hypertensive disorders, magnesium sulfate remains central for seizure prophylaxis. Diagnostic imaging should not be delayed, as maternal stabilization takes priority, with appropriate dose-minimization strategies [17]. Overall, optimal care requires a multidisciplinary approach and careful adaptation of standard stroke therapies to the unique physiological and safety considerations of pregnancy. In this context, the use of dual antithrombotic therapy during pregnancy is approached with caution due to limited safety data and the risk of maternal and fetal bleeding. As noted by van Alebeek et al [18], combination therapy with anticoagulants and antiplatelet agents is generally not recommended routinely, except in highly selected cases where maternal benefit clearly outweighs the risks. When antithrombotic therapy is indicated, a single-agent strategy is preferred, most commonly low-dose aspirin for secondary prevention or heparin-based anticoagulation for specific indications such as cardio-embolism or CVST. Dual therapy may be considered in exceptional high-risk situations (e.g., mechanical heart valves) but requires close monitoring due to the increased risk of hemorrhage, including obstetric bleeding. Overall, management prioritizes minimizing fetal exposure and maternal complications, favoring the safest effective regimen over routine combination therapy [18, 19]. Perelli et al [20] address the use of contrast agents during pregnancy, emphasizing that they should be reserved for clinically essential situations. Iodinated contrast agents, commonly used in CT imaging, cross the placenta and may affect fetal thyroid function, although no definitive teratogenic effects have been demonstrated. Gadolinium-based agents used in MRI also cross the placenta and have shown potential toxicity in animal studies; therefore, their use is generally avoided unless strictly necessary. Ultrasound contrast agents are considered safer due to minimal systemic absorption [21]. Overall, contrast agents are not absolutely contraindicated in pregnancy but should be limited to urgent or essential diagnostic indications with appropriate precautions [22]. Table 1 summarizes the key concepts of treatment.

**Table 1:** Key Concepts of Treatment

Aspect	Key points
Incidence	About 10–30 per 100,000 pregnancies; highest in peripartum/postpartum
Initial step	Immediate neuroimaging (noncontract CT); do not delay
IV thrombolysis (tPA)	Within 4.5 h; pregnancy not absolute contraindication; individualized risk–benefit
Mechanical thrombectomy	Indicated for large-vessel occlusion; safe in selected pregnant patients
Anticoagulation (acute phase)	Not recommended (increase hemorrhagic transformation risk)
Secondary prevention	Antiplatelet therapy after acute phase
Supportive care	Control BP, glucose (140–180 mg/dL), normothermia, avoid hypotonic fluids
Special etiologies	Manage CVST, dissection, RCVS per standard guidelines
Core principle	Treat as standard stroke with pregnancy-specific considerations

IV: intravenous; tPA: tissue plasminogen activator; CT: computed tomography; BP: blood pressure; CVST: cerebral venous sinus thrombosis; RCVS: reversible cerebral vasoconstriction syndrome.

The Canadian Stroke Best Practice Consensus Statement states that pregnancy should not exclude patients from intracranial mechanical thrombectomy when acute ischemic stroke due to large-vessel occlusion is present. Rapid maternal reperfusion is critical for both maternal and fetal outcomes and that concerns regarding fetal radiation exposure should not delay treatment, as exposure during cerebral angiography and thrombectomy is generally below harmful thresholds when dose-reduction strategies, including abdominal shielding and minimized fluoroscopy time, are used. Advanced imaging with CT/computed tomography angiography (CTA) or MRI/magnetic resonance angiography (MRA) is considered acceptable when clinically necessary. The statement also highlights the importance of multidisciplinary management and careful maintenance of maternal hemodynamic stability and uteroplacental perfusion during the procedure [23].

Aaron et al [24] described the successful use of mechanical thrombectomy with the Penumbra system in a pregnant woman presenting with acute ischemic stroke caused by large-vessel occlusion. Owing to severe neurological deficits and concerns regarding thrombolysis, endovascular treatment was performed with careful radiation-reduction measures to minimize fetal exposure. The authors supported mechanical thrombectomy as a feasible and potentially safe reperfusion strategy in selected pregnant patients [24].

The review of El-Refai [25] emphasized the importance of maintaining maternal oxygenation, hemodynamic stability, and uteroplacental perfusion during neurointerventional procedures, while avoiding hypotension, hypoxia, and significant fluctuations in intracranial pressure. Careful selection between conscious sedation and general anesthesia (GA) should be individualized according to neurological status, airway protection, and procedural complexity. The author underlined the need for multidisciplinary coordination and fetal safety considerations, including minimizing radiation exposure and optimizing maternal physiological stability throughout endovascular treatment [25].

It is recently showed that pregnancy and especially the puerperium increase stroke risk compared with non-pregnant women of similar age. Ischemic stroke was slightly more common than hemorrhagic stroke, while hypertensive disorders of pregnancy were identified as the major risk factor [26].

Swartz et al [27] reported that pregnancy and the puerperium are associated with a higher stroke risk compared with non-pregnant women of similar age, with an overall incidence of approximately 30 per 100,000 pregnancies. Risk was greatest during the third trimester and postpartum period. Ischemic stroke was the most common subtype, followed by intracerebral hemorrhage and cerebral venous thrombosis, while hypertensive disorders of pregnancy represented the major associated risk factor [27].

Leffert et al [28] demonstrated that pregnancy and the postpartum period increase the risk of hemorrhagic stroke compared with non-pregnant women of similar reproductive age, largely due to pregnancy-specific factors such as preeclampsia/eclampsia and hypertensive disorders. Although ischemic stroke is generally more common during pregnancy, hemorrhagic stroke carries substantially higher maternal morbidity and mortality, with increased rates of intensive care admission, prolonged hospitalization, and death. The postpartum period was identified as the highest-risk phase for hemorrhagic complications [28].

Other authors reported that intracerebral hemorrhage during pregnancy is rare but associated with high maternal mortality and severe morbidity. Pregnancy and the postpartum period were identified as important risk periods compared with non-pregnant women, particularly in the presence of hypertensive disorders such as preeclampsia/eclampsia. The study highlighted that hemorrhagic stroke, although less common than ischemic stroke overall, carries substantially worse maternal outcomes [29].

In our case, the patient was a 46-year-old pregnant woman who presented with severe disabling neurological deficits and a high anticipated risk of permanent functional impairment. Although DWI/ADC restriction was already evident on MRI, the infarct core appeared limited, and the overall clinical–radiological evaluation suggested the presence of potentially salvageable tissue. Perfusion imaging was not performed because of pregnancy-related considerations and the desire to avoid additional imaging exposure and contrast-related procedures.

The role of thrombectomy in distal and medium-vessel occlusions remains controversial following the recent ESCAPE-MeVO, DISTAL, and DISCOUNT trials, which questioned the routine use of endovascular treatment in these settings [30–32]. However, these studies included heterogeneous patient populations, and proximal or clinically dominant M2 occlusions may still represent a subgroup in whom thrombectomy could be considered reasonable, particularly in young patients presenting with severe disabling deficits.

Considering the patient’s young age, pregnancy, disabling clinical presentation, and the high likelihood of substantial long-term disability without reperfusion therapy, the multidisciplinary stroke team considered mechanical thrombectomy to be justified on an individualized basis despite the distal occlusion location and the presence of early ischemic MRI changes.

No significant stenosis of the cervical or intracranial arteries was identified, effectively excluding an atherothrombotic etiology. A thorough cardiac evaluation, including electrocardiogram (ECG), rhythm monitoring, and both transthoracic and transesophageal echocardiography, revealed no cardioembolic source, with no evidence of a patent foramen ovale or other structural cardiac abnormalities. The infarction itself was not consistent with a lacunar pattern, as it involved a large-vessel territory (e.g., M2), further guiding the differential diagnosis away from small-vessel disease. Additional investigations did not identify alternative specific causes, such as arterial dissection or thrombophilia conditions, including antiphospholipid syndrome. The only identifiable risk factor was pregnancy, a recognized hypercoagulable state; however, no direct etiological mechanism could be established. Consequently, after a systematic and comprehensive exclusion of all known causes, and in the absence of a clearly defined etiology, the event was classified as a cryptogenic stroke as the literature reports [33].

### Anesthetic choice for mechanical thrombectomy

The choice of anesthesia during endovascular thrombectomy significantly influences procedural safety and outcomes. In clinical practice, the decision is typically between GA and MAC, each with specific advantages and limitations [34]. GA provides airway protection, patient immobility, and optimal procedural conditions, particularly in patients with low Glasgow Coma Scale scores or technically complex procedures, although it may increase hemodynamic instability and ventilator-related complications [35]. In contrast, MAC enables faster procedural initiation and continuous neurological assessment but carries a risk of patient movement and possible conversion to GA in 9–16% of cases [36, 37]. Randomized trials and meta-analyses have generally demonstrated comparable neurological outcomes between GA and MAC during thrombectomy [38–44].

In pregnancy, anesthetic management requires additional consideration of maternal physiology and fetal safety. Pregnancy-related respiratory, cardiovascular, and hypercoagulable changes may increase anesthetic risk and influence drug pharmacokinetics [45]. Unlike non-pregnant patients, management must additionally consider fetal exposure during neuroimaging and fluoroscopy-guided thrombectomy, radiation protection strategies, gestational age, fetal monitoring, and the potential effects of anesthetic and vasoactive agents on uteroplacental perfusion [46]. Attention should therefore focus on maintaining maternal oxygenation, stable hemodynamics, left uterine displacement when appropriate, and avoidance of maternal hypotension or hypoxemia that could compromise fetal well-being. When feasible, MAC is often preferred because it may reduce the risks associated with intubation, maternal hypotension, and impaired uteroplacental perfusion [47]. Potentially teratogenic agents, including benzodiazepines and sugammadex, should be avoided whenever possible [48, 49]. Nevertheless, GA remains necessary in selected situations, and anesthetic management should be individualized through multidisciplinary collaboration, balancing maternal neurological needs with fetal well-being [50]. Recovery following monitored anesthesia care can still be stressful for patients and should occur smoothly in a monitored environment. As recovery often begins in the operating room, patients may arrive in the recovery unit at risk of airway compromise, shivering, agitation, delirium, pain, nausea and vomiting, hypothermia, and hemodynamic instability [51]. This phase is particularly important in pregnant women after mechanical thrombectomy, where close monitoring is essential to ensure maternal neurological recovery and fetal well-being.

### Conclusions

This case demonstrates that urgent mechanical thrombectomy can be safely and effectively performed in mid-pregnancy, even in advanced maternal age, achieving excellent maternal and fetal outcomes. It underscores the importance of rapid recognition, prompt neuroimaging, and a multidisciplinary approach when managing acute ischemic stroke during pregnancy. Pregnancy should not automatically preclude guideline-recommended reperfusion therapies, but careful individualized planning including consideration of anesthesia, maternal hemodynamics, and fetal safety, is essential. Reporting such cases contributes valuable evidence to guide clinical decision-making in this rare but high-risk scenario and supports expanding the use of endovascular interventions for pregnant patients with large-vessel occlusion.

### Learning points

Acute ischemic stroke during pregnancy, although rare, requires rapid diagnosis and timely reperfusion therapy, as maternal stabilization directly impacts fetal outcomes.

Mechanical thrombectomy can be safely performed in selected pregnant patients with large-vessel occlusion, offering favorable neurological and obstetric outcomes.

Management should follow standard stroke protocols while incorporating pregnancy-specific considerations, including imaging safety, anesthesia choice, and medication use.

A multidisciplinary approach is essential to optimize both maternal and fetal outcomes in this complex clinical scenario.

## Data Availability

The authors declare that data supporting the findings of this study are available within the article.
